# Comment on Aşır et al. Investigation of Vitamin D Levels in Men with Suspected Infertility. *Life* 2024, *14*, 273

**DOI:** 10.3390/life14070913

**Published:** 2024-07-22

**Authors:** Hiba Abid, Kainat Mehmood, Areeba Abid, Eisha Abid

**Affiliations:** Sindh Medical College, Jinnah Sindh Medical University, Karachi 75510, Pakistan; kainatmehmood133@gmail.com (K.M.); areebaabid0101@gmail.com (A.A.); eishaabid1018@gmail.com (E.A.)

We have reviewed the article “Investigation of Vitamin D Levels in Men with Suspected Infertility” by Fırat Aşır [1]. This study provides promising insights, and the author’s efforts are to be cherished, as it contributes to the investigation of the impact of Vitamin D deficiency on sperm parameters in men with suspected infertility. It also successfully demonstrates the correlation between vitamin D and sperm motility as well as testosterone. Moreover, the study also highlights the presence VDRs in reproductive organs which play vital roles in influencing male fertility. However, molecular details are yet to be discovered. We concur with all of the statistical findings and details provided in the study. However, it would be a privilege to append a few points to strengthen the study’s findings.

Initially, the single-center database study raised various considerations about the study’s validity. From December 2015 to December 2018, a multicenter study was conducted in the United States which incorporated data from nine hospitals, yielding significant findings [2]. These findings [2] contradict those of the study conducted by Fırat Aşır [1]. Furthermore, existing studies are also marked by considerable diversity in the study design, populations under investigation, vitamin D threshold values, and adjustments for potential confounding factors like age, sunlight exposure, and BMI [3]. Therefore, this study should have addressed the varying degrees of UV exposure and its impact on the prevalence of vitamin D deficiency in different regions of the world [4]. The global prevalence of vitamin D deficiency, influenced by variations in sunlight exposure, remains a critical health issue in 2024. According to recent databases, around 1 billion people worldwide are affected [5,6,7]. 

This study [1] undoubtedly has widened exclusion criteria and very precise inclusive criteria, which actually plays a key role in enhancing the validity of the results. The authors strongly emphasized the correlation between demographic parameters and vitamin D. However, there was a significant need to address the dietary habits of these participants, as dietary factors (e.g., specific food groups, nutrients, and nutritional supplements) have a major influence on both female and male reproductive functions [8], raising a consideration about the differences in fertility between vegan and non-vegan men, as vitamin D is considered as a significant concern of a vegan diet [9].

Additionally, the study does not address the environmental factors such as exposure to endocrine-disrupting chemicals (EDCs), which can affect fertility of men and may be correlated with vitamin D levels. Such an omission could have a profound impact on the authenticity of this research.

An individual is surrounded by EDCs every day, such as bisphenol A (BPA), phthalates, polychlorinated biphenyls (PCBs), dioxins, perfluorinated chemicals (PFCs), etc. EDCs are found in hydraulic fluids, printing inks (PCBs), receipts (BPA), raincoats (phthalates), polyvinyl items like toys, air fresheners, cleaning agents (phthalates), smoke from burning wood (dioxins), and even in soils and plants that contain pesticides. Our diets also contribute to EDC exposure. Vegetables, fruits, green tea, chocolate, and red wine all contain phytoestrogens, which are a certain kind of EDC. Apart from causing infertility at low doses, EDCs are also associated with premature puberty and other health problems [10].

However, the study’s failure to acknowledge these prevalent environmental factors would result in inadequate or incorrect conclusions about fertility as well as vitamin D levels.

Acknowledging the multifaceted influences of the factors mentioned may significantly amplify the authenticity and the validity of the study, giving a clearer understanding about the impact of vitamin D levels and male infertility.
